# Associations between Pet Ownership and Frailty: A Systematic Review

**DOI:** 10.3390/geriatrics5040089

**Published:** 2020-11-09

**Authors:** Gotaro Kojima, Reijiro Aoyama, Yu Taniguchi

**Affiliations:** 1 Department of Research, Dr. AGA Clinic, Tokyo 105-0004, Japan; 2 Department of Chinese and Bilingual Studies, The Hong Kong Polytechnic University, Hung Hom, Hong Kong; reijirou2000@gmail.com; 3 Center for Health and Environmental Risk Research, National Institute for Environmental Studies, Tsukuba 305-8506, Japan; taniguchi.yu@nies.go.jp

**Keywords:** frailty, frail elderly, pet, systematic review

## Abstract

Frailty is defined as a state of increased vulnerability due to age-related decline in reserve and function across multiple physiological systems. Increasing physical activity level is considered to be a measure to counteract frailty. Some studies have indicated that pet owners are more engaged in physical activity than non-owners. We conducted a systematic review regarding associations between pet ownerships and frailty among community-dwelling older adults and critically assessed the findings. PubMed was searched in April 2020 according to the Preferred Reporting Items for Systematic Review and Meta-Analysis (PRISMA) guidelines for cross-sectional or prospective studies examining associations between pet ownership and frailty in community-dwelling older adults with a mean age of 60 or above. A supplementary search was done using Google Scholar. Identified articles were reviewed by two investigators independently and assessed for methodological quality. The search identified 48 studies, among which three studies (two cross-sectional and one prospective) were included in this review. These studies suggested that pet ownership may be associated with a lower risk of frailty. This systematic review found only a limited amount of relevant research. More research is needed to establish the link between pet ownership and frailty as well as healthy aging and well-being.

## 1. Introduction

Global population aging is progressing at an unprecedented rate due to declining fertility rates and increasing life expectancy in most countries [1]. Worldwide, the percentage of older adults aged 65 or over was 9% in 2019, however, it is projected to increase up to 16% by 2050 [1]. The most problematic expression of aging is a clinical condition of frailty. This condition is one of the common geriatric syndromes affecting older adults and is theoretically defined as a state of increased vulnerability due to age-related decline in reserve and function across multiple physiological systems [2]. More and more individuals are affected as they get older, and a systematic review showed that pooled prevalence of frailty in those aged 65 or older is 10%, although the prevalence of individual studies varied in part due to a variety of definitions [3,4]. With the number of older people projected to grow worldwide, more older adults are expected to suffer from frailty in the coming decades. Previous studies have shown that compared with non-frail counterparts, frail older adults are more likely to experience a wide range of negative health outcomes, including falls [5], fractures [6], hospitalization [7], institutionalization [8,9], dementia [10], disability [11], poor quality of life [12], and premature death [13,14]. Given the expected high occurrence and devastating impacts of frailty, the syndrome has been recognized as a major challenge and priority for public health, requiring attention from healthcare professionals as well as researchers and policymakers [15].

For these reasons, frailty has received considerable scientific attention in recent decades, leading to a growing body of evidence on various interventions to prevent, delay, or reverse frailty and its related outcomes [16,17]. Although the most effective intervention has yet to be determined, one of the most promising ones is increasing physical activity levels. Insufficient physical activity increases risks of non-communicable diseases, such as cardiovascular diseases, cancer, diabetes, and premature death [18]. Previous research has shown beneficial effects of regular physical activity for physical, mental, and cognitive health in all age groups, including older adults [18,19]. Despite the well-known health benefits, physical activity levels tend to diminish as people age, and older adults spend more time engaging in sedentary behaviors [20].

Some studies have indicated that pet owners are more engaged in physical activity than non-owners [21,22,23], therefore pet ownership may be associated with reduced risk of frailty. Other factors associated with pet ownership include a favorable cardiovascular risk profile, such as lower systolic blood pressure or cholesterol levels, and improved mental well-being, such as less mental stress, loneliness, or depression [21,22,23,24]. These beneficial aspects of pet ownership could contribute to decreasing the risk of developing frailty or reversing already developed frailty status. Therefore, the objectives of this review were to perform a systematic review of the literature to identify currently available evidence on associations between pet ownerships and frailty among community-dwelling older adults, and to critically assess the findings of the relevant articles.

## 2. Materials and Methods

### 2.1. Search Strategy

A systematic search of the literature using PubMed was performed by one investigator (GK) in April 2020 within a time frame from 2000 to April 2020, as the most commonly used frailty phenotype criteria was first published in 2001 [25]. A protocol was created a priori based on the Preferred Reporting Items for Systematic Review and Meta-Analysis (PRISMA) statements [26] and submitted to PROSPERO for registration (https://www.crd.york.ac.uk/prospero/display_record.php?RecordID=182,313). The search strategy with Medical Subjective Heading (MeSH) and text terms was as follows: (“pet” OR “pets” OR “dogs (MeSH)” OR “dog” OR “dogs” OR “cats (MeSH)” OR “cat” OR “cats” OR “companion animal”) AND (“Frailty (MeSH)” OR “frailty” OR “Frail Elderly (MeSH)”). Titles, abstracts, and full texts were screened and examined for eligibility independently by two investigators (G.K. and Y.T.). Reference lists of relevant articles were reviewed for additional studies. A supplementary literature search was conducted with the same research terms using Google Scholar (https://scholar.google.co.jp/) in September 2020, which found no additional studies to be included.

### 2.2. Study Selection

Inclusion criteria comprised any studies that provided observational data on cross-sectional or prospective associations between pet ownership and frailty among older adults with a mean age of 60 years or above. Any studies using a sample of selected populations, such as patients with a specific disease (patients with Parkinson’s disease) or people in a specific condition (institutionalized individuals) were not considered. Frailty was to be defined using criteria validated in multiple settings and populations, such as the frailty phenotype [25]. Randomized controlled trials, reviews, editorials, conference abstracts, and dissertations were not considered. Corresponding authors were contacted if additional information was needed. 

### 2.3. Data Extraction

Data were extracted from each included study regarding first author, cohort name, if any, publication year, location, sample size, proportion of female participants, mean age, age range, frailty criteria, study design, and findings.

#### Methodological Quality Assessment

Cross-sectional studies were assessed for methodological quality using the 8-item Joanna Briggs Institute Critical Appraisal Checklist for Analytical Cross-Sectional Studies [27]. Methodological quality of prospective studies was evaluated using the 9-item Newcastle-Ottawa scale for cohort studies [28]. Adequate methodological quality was defined as having a score of more than half of the full potential score.

### 2.4. Statistical Analysis

If the same effect sizes of frailty risk according to pet status were available from two or more studies, a meta-analysis would be attempted to synthesize pooled risk estimates.

## 3. Results

### 3.1. Selection Processes

**Figure 1:**
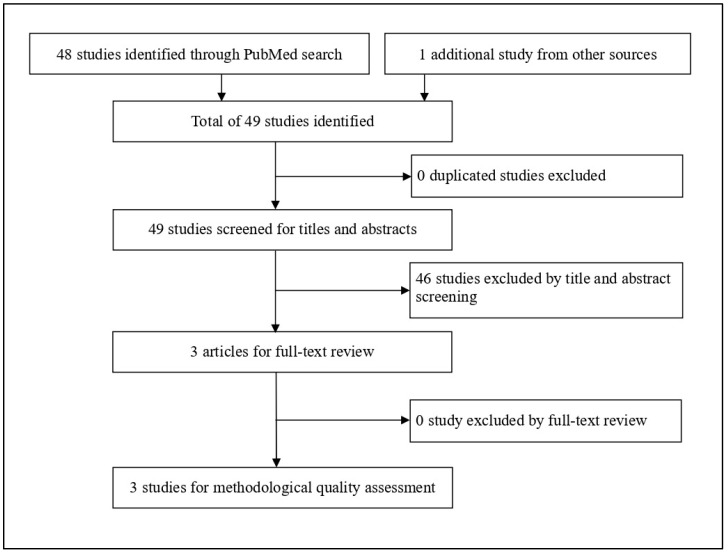
Flow chart of systematic literature review.

The PubMed search identified 48 studies; one additional study was found by reviewing reference lists of relevant articles. Forty-five studies were excluded based on the title and abstract screening, and no study was excluded based on the full-text review. This left three studies [29,30,31] that fulfilled the inclusion criteria for this review. The flow chart of the literature search is shown in Figure 1.

### 3.2. Study Characteristics

Three studies (two cross-sectional [30,31] and one longitudinal [29]) were included in this review (Table 1). All three studies are from Japan. Two of them [30,31] used data of Japanese older men and women from the same cohort, the Ota Genki Senior Project, a community-wide intervention trial in one of the wards in Tokyo, Japan [32]. The project data for frailty and pet ownership were collected from mailed self-administered questionnaires [32]. Frailty was defined using the Kaigo-Yobo Checklist [14,33], and pet ownership items included current, past, or never experience living with a dog or cat [30,31]. The other study used modified Cardiovascular Health Study criteria to define frailty, and gathered information on pets using a questionnaire [29].

The first study [30] cross-sectionally examined the prevalence of frailty among current, past, or never dog/cat owners, which were 22.3%, 23.4%, and 24.7%, respectively. Although not adjusted, the calculated odds ratio (OR) of being frail for current dog/cat owners was significantly lower than that of never owners (OR = 0.88, 95% confidence interval (CI) = 0.77–1.00), while there was no significant difference in frailty risk between past dog/cat owners and never owners (OR = 0.93, 95%CI = 0.84–1.03).

Another cross-sectional study examined physical frailty, depressed mood, and psychological frailty, defined as having both physical frailty and depressed mood according to the authors, in relation to various lifestyle activities and incident disability [29]. One of the lifestyle activities was whether or not a participant was taking care of grandchildren or pets. Based on the data provided in this paper, those taking care of grandchildren or pets had a 40% lower risk of being frail compared with those who were not (calculated odds ratio = 0.60, 95%CI = 0.47–0.76) [29].

The other study [31] prospectively followed the cohort for incident frailty over 2 years. Compared with never dog/cat owners, current and past owners had lower odds of developing frailty, with statistical significance reached only in past owners (OR = 0.84, 95%CI = 0.71–0.98) but not in current owners (OR = 0.87, 95%CI = 0.69–1.09). In stratified analyses, incident frailty risk was assessed based on ownership of dog and cat separately. Past dog owners had a significantly lower risk of developing frailty (OR = 0.82, 95%CI = 0.69–0.99) while current dog owners did not (OR = 0.81, 95%CI = 0.62–1.13), compared with never owners. There were no significant differences in incident frailty risks in current and past cat owners from those in never cat owners.

All studies had more than half of the full scores on methodological quality assessment tools and were considered to have adequate methodological quality (Table 2 and Table 3). Although odds ratios of being frail were available from two cross-sectional studies, a meta-analysis was not possible because the pet-related factors were not similar (ownership of dog/cat [30] vs. whether or not the participants were taking care of grandchildren or pets [29]).

## 4. Discussion

This review identified three articles examining associations between pet ownership and frailty in community-dwelling older adults. Although a meta-analysis was not possible, some of the analyses from the included studies suggest that pet ownership may be associated with a lower risk of frailty. No studies showed that pet ownership was associated with a high risk of frailty.

An online survey conducted on 27,000 people aged 15 and older in 22 countries showed that more than half of the respondents in the surveyed countries had at least one pet (43% no pets, 33% dog, 23% cat, 12% fish, 6% bird, and 6% others) [34]. The popularity of pet ownership has spurred increasing research into human-animal interactions in recent years, which has generated a growing body of evidence of various benefits of pet ownership for humans [35]. In particular, the exploration of potential roles that pets may play in healthy aging is one of the topics investigated in a number of recent studies [35]. Multiple studies have shown that pet ownership may be beneficial for well-being and for preserving physical, mental, and social functions in older adults, and may also contribute to their sustaining independence [36]. For example, pet owners, particularly dog owners, may go out more frequently and engage in social interactions with other pet owners. The companionship of a pet may comfort and decrease levels of stress or depressive mood [37]. 

Studies have demonstrated that older pet owners are significantly more likely to walk, have higher levels of physical activity, avoid sedentary behaviors, and retain activities of daily living (ADL) levels [36]. These benefits of pet ownership could play a key role in decreasing frailty risk and support the findings of studies included in this review [29,30,31].

In addition to the impacts of pet ownership on physical health, it was suggested that human-animal interactions could have potential effects on psychosocial well-being. Effects of animal-assisted activities and therapy among older adults in different settings have been well-documented. Older adults in long-term care or assisted living facilities have been a particular focus of studies and were found to benefit from contact with animals, with such effects as decreased depressive symptoms and loneliness [24]. A meta-analysis including five studies showed that animal-assisted activities/therapy significantly decreased depressive symptoms (standard difference = 0.61, 95% confidence interval = 0.03–1.19) [37]. Regarding anxiety, most studies that examined the effects of animals on anxiety showed significantly reduced anxiety levels with animals, especially under stressful circumstances [24]. Furthermore, pet ownership could play a role as a catalyst in facilitating interpersonal social interactions and enhancing social networks. One study showed that being accompanied by a dog was significantly associated with more frequent social interactions than otherwise [38]. As depression and poor social profiles are known risk factors of frailty [39,40], pet ownership may be associated with decreased frailty risks via these pathways.

There are some limitations to this study. First, only three studies were identified regarding associations between pet ownership and frailty. Second, a meta-analysis was not possible, partially due to the scarcity of the related studies. Third, the pet data provided by the included studies were rather crude, and data on specific types of pets, such as dog, cat, bird, or reptile, were not available. Despite these limitations, this review has multiple strengths, the major one being its robust methodology, which was in accordance with PRISMA statements. A comprehensive and reproducible search strategy was used for a literature search in multiple databases with a combination of MeSH and text terms. The titles, abstracts, and full texts of the identified studies were screened by two investigators independently with a pre-created protocol. The included studies were examined for methodological quality using widely used assessment tools.

## 5. Conclusions

This systematic review focused on the association between pet ownership and frailty risk. We located only a limited amount of research fulfilling the inclusion criteria. Given the plausible health benefits of pets for older adults, more research is needed to establish the link between pet ownership and frailty as well as healthy aging and well-being.

## Figures and Tables

**Table 1:** Summary of included studies on associations between pet ownership and frailty in older people.

Author/Study	Year	Location	Sample Size	Female (%)	Mean Age (Range)	Frailty Criteria	Study Design	Findings
Taniguchi et al. Ota Genki Senior Project	2018	Japan	11,233	51.5%	− (75–84)	KYCL	Cross-sectional	-Percentage of frailty in current, past, and never dog/cat owners was 22.3%, 23.4%, and 24.7%, respectively, with no significant differences (*p* = 0.063) -Calculated OR of being frail (never owner as reference) cOR = 0.88 (95%CI = 0.77–1.00) for current dog/cat owners cOR = 0.93 (95%CI = 0.84–1.03) for past dog/cat owners
Shimada et al. NCGG-SGS	2019	Japan	4126	53.6%	71.7 (≥65)	mCHS	Cross-sectional	-Percentage of frailty in those who were taking care of grandchildren or pets and those who were not was 5.4% and 8.7%, respectively -Calculated OR = 0.60 (95%CI = 0.47–0.76) of risk of being frail for those who were taking care of grandchildren or pets compared with those who were not
Taniguchi et al. Ota Genki Senior Project	2019	Japan	6197	53.6%	73.6 (≥65)	KYCL	Longitudinal (2-year follow-up)	-Mixed-effect logistic regression models for incident frailty adjusted for age, gender, household size, equivalent income, history of stroke, food variety, Geriatric Depression Scale 5 score, alcohol status, and smoking status (never owner as reference) aOR = 0.87 (95%CI = 0.69–1.09) for current dog/cat owners aOR = 0.84 (95%CI = 0.71–0.98) for past dog/cat owners

95%CI = 95% confidence interval; aOR: adjusted odds ratio; cOR: calculated odds ratio; KYCL: Kaigo–Yobo Checklist; mCHS: modified Cardiovascular Health Study criteria; NCGG-SGS: National Center for Geriatrics and Gerontology-Study of Geriatric Syndromes.

**Table 2:** Methodological quality assessment with the Joanna Briggs Institute Critical Appraisal Checklist for Analytical Cross-Sectional Studies.

Author/Year	Inclusion	Setting	Exposure	Measurement	Confounders	Strategies for Confounders	Outcome	Statistical Analysis	Total
Taniguchi 2018	1	1	1	1	1	1	1	1	8/8
Shimada 2019	1	1	0	1	1	1	1	1	7/8

**Table 3:** Methodological quality assessment with the Newcastle-Ottawa Quality Assessment Scale for cohort studies.

Author/Year	Representativeness	Non-Exposed Group	Ascertainment of Exposure	Absence of Outcome at Baseline	Controlling for Important Factors	Controlling for Additional Factors	Ascertainment of Outcome	Length of Follow-Up	Adequacy of Follow-Up	Total
Taniguchi 2019	1	1	0	1	1	1	0	1	1	7/9
